# A Case of a Novel MAGED2 Mutation Resulting in Non-transient Bartter’s Syndrome in an Adult Female

**DOI:** 10.7759/cureus.38681

**Published:** 2023-05-07

**Authors:** Isam Albaba, Sharmeen Azher, Swati Mehta, Geovani Faddoul

**Affiliations:** 1 Internal Medicine, Albany Medical Center, New York, USA; 2 Internal Medicine, Baystate Medical Center, Springfield, USA; 3 Nephrology, Internal Medicine, Albany Medical Center, New York, USA

**Keywords:** novel mutation, mage-d2 mutation, type v bartter syndrome, transient bartter syndrome, bartter syndrome

## Abstract

Bartter’s syndrome (BS) is a disorder caused by a group of rare mutations that result in defective salt reabsorption in the thick ascending loop of Henle. BS is characterized by salt wasting, hypokalemia, and metabolic alkalosis, among other abnormalities. A MAGE-D2 mutation results in an X-linked form of BS. It results in a transient antenatal presentation that is observed to completely resolve by early infancy, usually occurring in males. We present a case of an adult female with intermittent recurrence of symptoms and metabolic derangements consistent with BS. She also has a family history of polyhydramnios and renal disease. Genetic testing later confirmed a novel MAGE-D2 mutation. Her atypical presentation emphasizes the heterogenous presentation of the different mutations and raises the possibility of persistence of abnormalities beyond infancy in mutations of the MAGE-D2 gene.

## Introduction

Bartter syndrome (BS) is a rare genetic disorder that results in impaired salt reabsorption in the thick ascending loop of Henle (TAL), the prevalence of which is 1 per 1,000,000 live births [1,2]. BS is classified into antenatal (types I, II IVa, IVb, and V) and classic forms with presenting ages of zero to five years (type III), which differ based on the protein involved and the onset of presentation [2]. Type V BS is caused by a myriad of mutations affecting melanoma-associated antigen D2 (MAGED2) protein function. New mutations are constantly being discovered [3]. In this paper, we report a novel *MAGED2 *pathogenic variant resulting in an atypical persistence of symptoms, rather than the more usual “transient antenatal” nature seen in Type V BS.

## Case presentation

A 43-year-old woman presented with hypokalemia, hypomagnesemia, and hypocalcemia. She had been recently hospitalized for severe hypokalemia with facial paralysis. She reported always being an avid salt eater as a child. Symptoms started in her early teens, with generalized edema and rapid alternating weight gain and loss. Oftentimes, she would experience palpitations, weakness, myalgia, and spasms. With age, she noticed her systolic blood pressure increasing slowly from a baseline of 90 to 120 mmHg. More recently, she had multiple visits to the emergency room due to vomiting and muscle weakness and and was found to have hypokalemia and hypocalcemia. Those episodes were mostly precipitated by exercising.

She had a history of pituitary adenoma and hypothyroidism, treated with cabergoline and Levothyroxine, respectively. She denied a history of nephrolithiasis, hematuria, or foamy urine. Family history was notable for a sister who died after birth at the age of two weeks after a pregnancy that was complicated by polyhydramnios and nephrocalcinosis. The direct cause of death is unknown. The patient has a daughter who is asymptomatic except for being an avid salt eater and having a pregnancy that was uneventful. Current medications include bromocriptine, ibuprofen, tramadol as needed, and daily potassium (20 mEq), levothyroxine, fluoxetine, calcium, and vitamin D.

Table 1 shows the laboratory workup for the patient. This showed normal sodium, low potassium despite supplementation, high bicarbonate, low magnesium, and normal urea, nitrogen, and creatinine levels. Both plasma renin activity and aldosterone levels were high. Urine studies showed a near-zero fractional excretion of sodium, high fractional excretion of potassium, and high fractional excretion of calcium. PTH levels were high most of the time they were checked over the course of 15 months. After counseling the patient, she made the decision to pursue genetic testing. Commercial testing was performed using next-generation sequencing. The test evaluates all the genes known to be associated with Bartter syndrome, including *AP2S1, BSND, CASR, CLCNKA, CLCNKB, CLND16, CLDN19, GNA11, HSD11B2, KCNJ1, MAGED2,* and *SLC12A1*. This showed a *MAGED2* X-linked heterozygous gene mutation (p.Pro268Leu; CCT>CTT) c.803 in exon 4. The lab reported that the variant identified was of uncertain significance since it was not observed in large population cohorts [4]. In silico analysis performed using Protein Variation Effect Analyzer (PROVEAN) [5] supported that this missense variant has a deleterious effect on protein structure and function. No mutations in the other evaluated genes were detected.

**Table 1:** Laboratory values PRA, plasma renin activity; PTH, parathyroid hormone; FeNa, fractional excretion of sodium; FeK, fractional excretion of potassium; FeCa, fractional excretion of calcium ^Labs drawn while patient on potassium, calcium, and vitamin D supplements *values were checked multiple times; the range shows the minimum and maximum values detected

	Value^	Reference normal ranges
Serum values		
Sodium (mmol/L)	137	135-145
Potassium (mmol/L)	3.4	3.6-5.2
Chloride (mmol/L)	97	96-106
Bicarbonate (mmol/L)	31	22-29
Magnesium (mg/dL)	1.6	1.7-2.2
Urea nitrogen (mg/dL)	19	6-24
Creatinine (mg/dL)	0.97	0.57-1.00
PRA (ng/mL/hour)	36	0.167-5.380
Aldosterone (ng/dL)	51.6	>3.0 – 23.2 while supine
PTH (pg/mL)	14.5-126.8*	15.0-65.0
Urine values		
Sodium (mEq/L)	11	-
Potassium (mEq/L)	74	-
Chloride (mEq/L)	15	-
Calcium (mg/dL)	3	-
FeNa (%)	0.03	<1.0
FeK (%)	6.76	1.5-6.4
FeCa (%)	0.1	<0.01

The patient was started on spironolactone and enalapril. Over the course of one year, electrolyte abnormalities improved but metabolic alkalosis persisted. She continued to have intermittent symptoms prompting several presentations to the ER, mainly driven by sweating during hot weather or exercise. Symptoms were better controlled during winter and when avoiding exercise.

## Discussion

This case of a 43-year-old female with BS has genetic testing confirming a previously unreported *MAGED2* mutation (c. 803 C>T p. P268L). Given that transient antenatal BS, by definition, resolves in the perinatal phase [6,7], the persistence of symptoms into adulthood in our case may be explained by an atypical phenotype of Type V BS caused by a pathogenic *MAGED2* variant.

**Figure 1:**
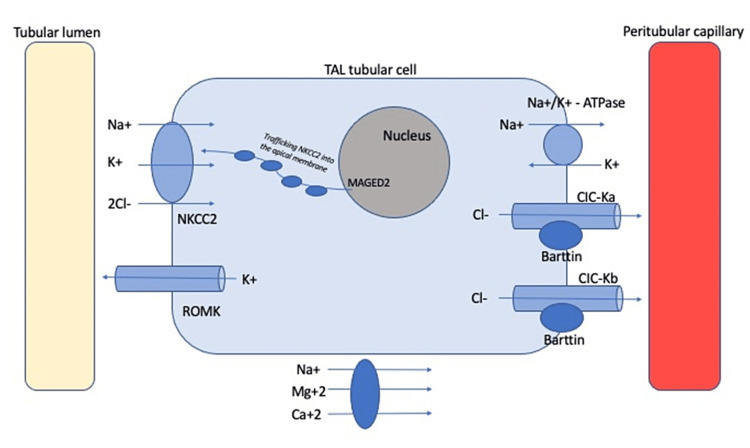
Protein channels involved in maintaining normal NKCC2 function TAL, thick ascending loop of henle; Na+, sodium; K+, potassium; Cl-, chloride; Mg+2, magnesium; Ca+2, calcium; NKCC, sodium-potassium co-transporter; ROMK, renal outer medullary potassium channel; MAGED2, melanoma-associated antigen D2 gene; Na+-K+ ATPase, sodium-potassium adenosine triphosphatase; CIC-K, voltage-gated chloride channel, K subtype Image Credits: I. Albaba

In normal physiology, sodium (Na+), potassium (K+), and chloride (Cl-) are co-transported through the NKCC2 transporter in the TAL, which is sensitive to loop diuretics [8]. While sodium is actively transported through a Na+/K+ ATPase, chloride exits the tubular cell via chloride channel subunits CIC-Ka and CIC-Kb [9]. Both chloride channel subunits require a functioning Barttin protein, found in the TAL as well as the inner ear [10]. Potassium recycling occurs via renal outer medullary K+ channels (ROMK), which is necessary for NKCC2 function, and this mechanism enables passive transport of Na+, K+, magnesium (Mg+2), and calcium (Ca+2) in the TAL [11]. The *MAGED2* gene is thought to be involved in the cytoplasmic trafficking of NKCC2 to the apical membrane of TAL as well as its activation [7]. Figure 1 illustrates the interaction of all these proteins together to maintain normal NKCC2 function.

The different types of BS result from different mutations that impair the function of proteins directly or indirectly interacting with the NKCC2 co-transporter. Type I results from mutations of gene SLC12A1 encoding the NKCC2 protein [8]. Type II results from mutations of gene KCNJ1 encoding the renal outer medullary K+ channels (ROMK) [11]. Type III is caused by mutations of gene CLCNKB in the chloride channel subunit CIC-Kb [9]. Type IV results from either dysfunctional Barttin protein in type 4a or CLC-Ka and CLC-Kb in type 4b [10]. Type V is a transient antenatal, X-linked recessive form of BS caused by mutations in *MAGED2* [3,6,7,11]. The abnormal *MAGED2* protein is believed to result in defective cytoplasmic trafficking and inactivation of NKCC2 [7]. Table 2 demonstrates the different mutations, defective proteins, and pathophysiologic effects for each type of BS.

**Table 2:** Types of BS with their associated mutations and defective protein channels BS, Bartter Syndrome; SCLC12A1, solute carrier family 12 member 1; NKCC2, sodium-potassium co-transporter 2; TAL, thick ascending loop of henle; KCNJ1, potassium inwardly rectifying channel subfamily J member; ROMK, renal outer medullary potassium channel; CLCNKB, chloride voltage-gated channel Kb gene; CIC-Kb, chloride voltage-gated channel Kb protein; BSND, Barttin CLCNK type accessory subunit beta gene; MAGED2, melanoma-associated antigen D-2 [3,6-12]

BS type	Mutated gene	Defective protein	Pathologic effect
I	SCLC12A1	NKCC2	Blocks Na-K-2CL cotranstport at TAL
II	KCNJ1	ROMK	Blocks K+ recycling that is necessary to maintain a K+ gradient for NKCC2 function
III	CLCNKB	CIC-Kb	Blocks basolateral Cl- transport that is necessary to maintain a Cl- gradient for NKCC2 function
IVa	BSND	Barttin	Defective Barttin protein impairs the function of CIC-Ka and CIC-Kb Chloride channels
IVb	CLCNKA/CLCNKB	CIC-Ka/CIC-Kb	
V	MAGED2	MAGE-D2	Defective trafficking of NKCC2 to the apical membrane

Impaired salt reabsorption in BS leads to upregulation of the renin-angiotensin-aldosterone system (RAAS). This causes increased cyclooxygenase-2 activity and prostaglandin production as an auto-regulatory mechanism, contributing to low blood pressure commonly observed in already volume-depleted BS patients [13,14]. Increased distal delivery of sodium is accompanied by the increased loss of potassium and hydrogen in the urine. Bicarbonate retention follows the same mechanisms causing alkalosis in settings of increased aldosterone release, hypokalemia, and hypochloremia [15]. Polyuria likely results from an impaired ability to concentrate urine, while nephrocalcinosis occurs in certain subtypes due to hypercalciuria [16].

The* MAGED2* protein interacts with G-alpha-S (GaS) and MDM2, which facilitates AMPc-dependent activation of the NKCC2 protein and salt reabsorption [17]. This process is impaired when the *MAGED2* gene is mutated. Vasopressin levels increase with age, which increases GaS activity and decreases reliance on *MAGED2* interaction. This can explain the transient nature of the disease. Another potential explanation is the resolution of tissue hypoxia in the fetal kidney that decreases endoplasmic reticulum-associated destruction of NKCC25. In one large French cohort of patients with BS, salt and water losses resolved by two to 18 months of age in 17 patients with *MAGED2* gene mutation, with discontinuation of COX-2 inhibitors and other medications. The mean follow-up was five years [3]. Since this mutation has only recently been described as a cause of BS, a longer period of follow-up is required to determine if presentation may recur. Furthermore, several mutations of *MAGED2* have all been grouped into this entity of “transient antenatal” BS. Studies have shown that there is extensive heterogeneity and variability in presentation [11,18]. It could be argued that this same heterogeneity may result in the persistence or recurrence of symptoms or electrolyte abnormalities in some patients like ours. Another explanation of the presentation is that this mutation is working with another mutation (two-hit theory) in a different gene causing the perpetuation of the Bartter phenotype through a non-resolution of the fetal hypoxia described by Seaayfan et al. [17].

Although the new variant of *MAGD2* detected in our patient is reported as “of uncertain significance”, in silico analysis (PROVEAN method) suggests it has effects on protein structure. Additionally, there are still numerous variant mutations of the *MAGED2* gene being reported in the literature, and there are likely more to be revealed in the future. Genetic testing of other members of the family is currently being pursued, albeit with financial challenges. If found to be positive, this will support the claim that her presentation is caused by this new *MAGED2* variant.

Transient antenatal BS is an X-linked recessive disorder and most cases reported in antenatal *MAGED2* gene mutations were male. Female carriers are usually asymptomatic carriers. In Legrand’s report, two of the patients were female and had a severe presentation, which was attributed to X-inactivation (random X-inactivation and unfavorably skewed X-inactivation). Hemizygous situations were described as well [3]. Our case is one of a heterozygous genotype. The presentation of polyhydramnios, nephrocalcinosis, and death early in life in the patient’s sister may be related to the same genetic mutation. If this is indeed the case, then X-inactivation (like the one described in Legrand’s series [3]) could be the culprit in the diametrically opposite phenotypes seen between sisters with the same mutation.

We opted to treat with spironolactone and angiotensin-converting enzyme inhibitors instead of the non-steroidal anti-inflammatory (NSAID) approach to avoid inducing NSAID nephropathy, which we saw in our experience in patients with Bartter’s syndrome who were treated with NSAIDs as children. The patient had stable renal function and her symptoms were better controlled.

## Conclusions

Type V BS is a transient antenatal X-linked form of BS. It can be caused by several different *MAGED2* gene mutations. In this case report, we describe the unusual phenotypic presentation in association with a newly detected *MAGED2* pathogenic gene variant. It highlights the phenotypic variability of different mutations of the same gene. In this example, the mutation may have resulted in the persistence of symptoms, unlike the usual transient BS that is usually seen with other *MAGD2 *gene mutations. Furthermore, the presentation in a female patient emphasizes that X chromosome inactivation is a potential factor in phenotype variability and the possibility of an X-linked mutation causing disease in a female carrier.
