# Correction: A Case of a Novel MAGED2 Mutation Resulting in Non-transient Bartter’s Syndrome in an Adult Female

**DOI:** 10.7759/cureus.c119

**Published:** 2023-05-23

**Authors:** Isam Albaba, Sharmeen Azher, Swati Mehta, Geovani Faddoul

**Affiliations:** 1 Internal Medicine, Albany Medical Center, New York, USA; 2 Internal Medicine, Baystate Medical Center, Springfield, USA; 3 Nephrology, Internal Medicine, Albany Medical Center, New York, USA

This article has been corrected at the request of the authors to remove a laboratory test accession number from the case presentation section. The accession number could be tied to a patient and result in breach of confidentiality. As a result it has been removed from the article. The authors regret that this was not discovered prior to publication.

